# Rhythm Across Species: Situating Human Speech in a Comparative Framework

**DOI:** 10.1111/nyas.70383

**Published:** 2026-08-31

**Authors:** Lara S. Burchardt, Ludger Paschen, Susanne Fuchs

**Affiliations:** ^1^ Humboldt Universität zu Berlin Berlin Germany; ^2^ Leibniz‐Zentrum Allgemeine Sprachwissenschaft Berlin Germany; ^3^ Universität Regensburg Regensburg Germany; ^4^ IMéRA and ILCB Marseille France

**Keywords:** animal communication, beat precision, cross‐species comparison, interonset interval, isochrony, speech rhythm

## Abstract

At the level of smaller linguistic units, such as words and syllables, rhythm is characterized by a high degree of heterogeneity between languages. This suggests that it is distinct from nonhuman animal communication, where underlying isochrony is often observed. In this study, we examine speech rhythm using units similar to those previously examined for nonhuman animals: interonset intervals (IOIs), that is, sound elements surrounded by silence. Surprisingly, IOI durations are relatively similar across a comprehensive sample of 48 languages from all inhabited continents, with a median of approximately 2 s. IOI beats are also relatively similar at around 0.5 Hz, and human speech fits between exemplary animal species in terms of rhythmic variability and beat precision, that is, the extent to which an isochronous beat model fits the sequence. By revealing cross‐linguistic regularities and comparing them to those of other animal species, our results help to position human speech within the broader spectrum of rhythmic vocal behavior observed in nature.

## Graphical abstract

We analyzed speech rhythms from 48 human languages using methods drawn from biology and linguistics. Interonset intervals across languages are surprisingly similar, with a median value of 2 seconds., despite substantial differences in linguistic structure and prosody. A comparative rhythm analysis shows that human speech falls within the range reported for selected birds, fish, and mammals, challenging the assumption that human speech rhythm is distinct.

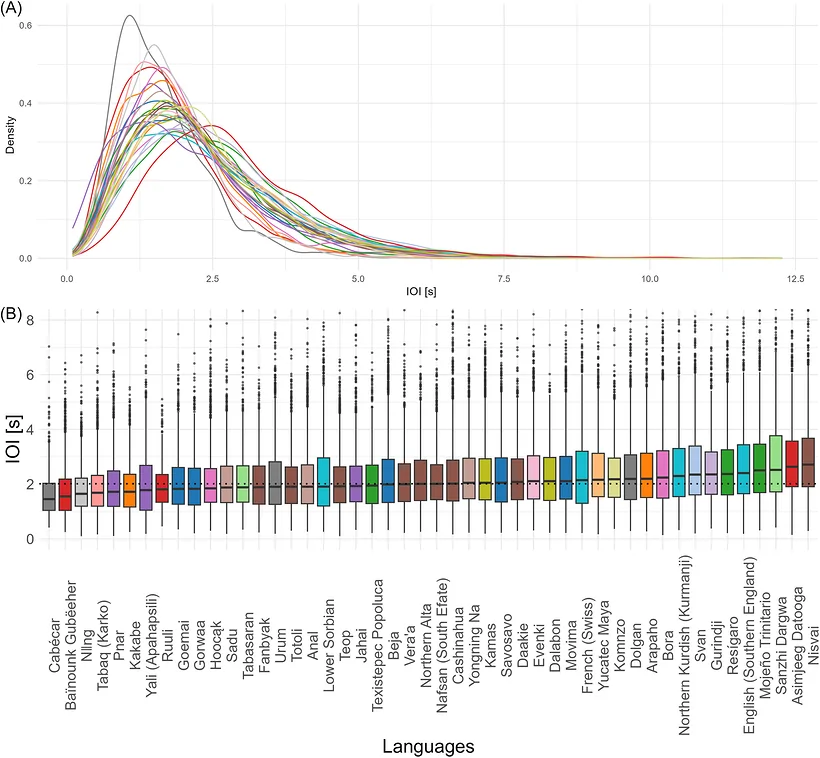

## 1 Introduction

### 1.1 Rhythm as a Fundamental Component of Communication

Rhythm is a fundamental component of communication across species. It organizes the temporal structure of events, enhances perceptual and cognitive processing, and enables predictions about forthcoming speech signals [1, 2, 3]. This temporal organization is not unique to human speech; it is a widespread property of biological signaling systems and provides a powerful comparative framework for understanding how timing is, on the one hand, shaped by certain biological properties and, on the other hand, carries meaning with regard to the individual and in interaction with others [4, 5, 6, 7, 8, 9, 10, 11].

**FIGURE 1:**
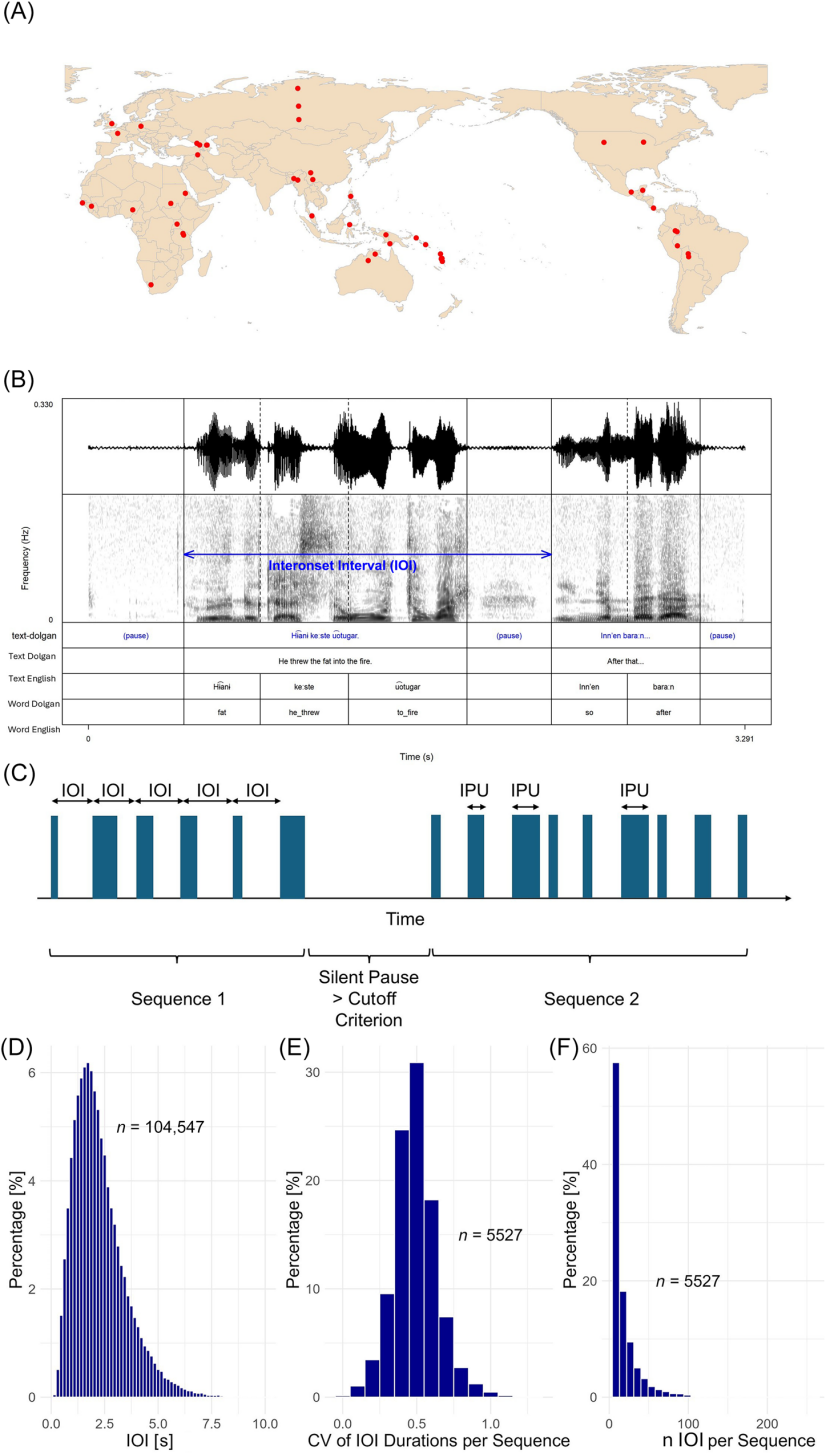
(A) Map showing the approximate locations of the 48 languages included in our study following Glottolog. (B) An illustration of an interonset interval (IOI), defined as spanning an interpausal unit (IPU) and a following speech pause, with an image of a recording from the Dolgan DoReCo dataset [63]. (C) A recording with two sequences, split because of a silent pause exceeding the 3.36 s threshold, with illustrations of IOIs and IPUs. (D–F) Results for various rhythm measures based on our data.

In this corpus‐based study, we focus on the production of rhythm using a descriptive approach and examine the temporal organization of human speech within a cross‐species context. Specifically, we analyze the durations of successive speech that is uninterrupted by pauses, called interpausal units (IPUs; Figure 1C; an overview of all definitions is presented in the Supporting Information, Table S1) [12], and interonset intervals (IOIs; see Figure 1C and Table S1), which include an IPU and a following silent pause (see Figure 1B and Table S1). These intervals provide a measure of the rhythmic organization of speech at a relatively slow temporal scale, comparable to those studied in nonhuman communication systems.

The main goal of our study is to investigate whether speech rhythms in humans align with the broader distribution of rhythmic behaviors observed across animal taxa, or whether it exhibits distinct species‐specific patterns. We think that it is important to locate human speech within vocalizing animal taxa as an important step for understanding the function and evolution of rhythmic behavior across species. Rather than focusing on linguistic rhythm categories (e.g., stress‐timed or syllable‐timed languages; see [13, 14, 15]) that might be human specific, we adopt a data‐driven perspective that treats timing itself as the primary observable dimension of communication. We operationalize timing as IOIs, a measure that is frequently used in descriptions of communicative rhythms in nonhuman animals. This measure is less vulnerable to the problem of theoretical mismatch, where concepts derived from human psychology and linguistics are uncritically applied to other species. Our main research question is as follows: Does rhythm in human speech production fit within a broader animal rhythm space regarding IOIs and related structural variability measures or is it different from nonhuman animals?

### 1.2 A Comparative Approach to Rhythm

A comparative approach across species allows us to situate human speech patterns within communicative timing phenomena in animal taxa. However, it is important that such an approach avoids relying on insider knowledge of human communication, that is, an anthropomorphic perspective (for further discussion, see [16]). Some studies have taken human linguistic frameworks to interpret nonhuman vocalizations. For example, Mann et al. [17] analyzed budgerigar song through a segment‐based lens inspired by human phonology, revealing patterns analogous to consonant‐ and vowel‐like elements. While their results are important for understanding segmental organization, applying linguistic terminology to nonhuman animals comes with its own risks, since comparative studies require common metrics and unified terminology. For example, terms like *syllable* have divergent meanings across linguistics and biology: in linguistics, a syllable refers to a unit of speech consisting of a sequence of one or several sounds, though its specific characteristics vary across languages; in biology, syllables are often defined as communicative expressions delineated by silent pauses [18, 19, 20].

We define rhythm in a way that is applicable to both humans and other species: rhythm is understood as a nonrandom, ordered, and recurrent alternation of events in time [21]. Isochrony is treated as an idealized model; intervals between events are equal. While perfect isochrony is rarely observed in natural signals, it provides a useful descriptor of a consistent temporal structure of approximately similar IOIs. A goodness‐of‐fit parameter (here beat precision [BP]) is then needed for assessing how closely real sequences approximate regular timing [22].

### 1.3 Rhythms in Nonhuman Communication

Rhythms in animal communication have been studied for decades, and the literature, especially on arthropod rhythms, is vast [11]. The field of research has recently experienced renewed momentum. In addition to several methods and opinion pieces that have been published in the last years [23, 24, 25, 26], new rhythm studies have appeared for species including zebra finches (*Taeniopygia guttata*) [4], bats [5, 18], rock hyraxes (*Procavia capensis*) [27], fish [9, 21], frogs [7], sperm whales (*Physeter macrocephalus*) [8, 25], gibbons [6], palm cockatoos (*Probosciger aterrimus*) [28], and the northern elephant seal (*Mirounga angustirostris*) [29], among others. Despite employing diverse methodologies, these studies consistently identify a pattern approximating isochrony to describe various sequence types. The findings suggest that rhythmic temporal organization may serve broad communicative functions such as coordination, attention alignment, or signal predictability.

While the overarching rhythmic structure appears similar across species, differences emerge in the specific frequency rates (e.g., beats per second) and the degree to which each species adheres to an underlying isochronous rhythm, that is, how well an isochronous model fits or how well a species keeps a beat, for example, as quantified with a goodness‐of‐fit parameter [4, 25, 30]. Through a combination of new methodological developments and efforts to find an evolutionary basis for communication rhythms, an increasing amount of knowledge about these rhythms can now be reasonably compared across species, enabling more general conclusions about their function, mechanisms, ontogeny, and evolution. Importantly, all these patterns have been described using data‐driven analyses that do not assume symbolic or linguistic representations, allowing meaningful comparison across species.

### 1.4 Rhythms in Human Communication

Human speech exhibits complex temporal structure shaped by linguistic, physiological, and cognitive constraints. Traditional typologies of rhythm that grouped languages into stress‐timed, syllable‐timed, or mora‐timed types [13, 14, 15] (for an explanation of terms, we refer to Table S1) have largely been replaced by quantitative descriptions of variability in segmental and prosodic timing [31]. Experimental studies have demonstrated that rhythm in human speech can provide expectancy cues for listeners [32] and play an important role in interactive communication [33, 34, 35]. Although an idealized isochronic rhythm has often been reported in nonhuman animals, empirical evidence for linguistic units below intonation units (IUs) (see next paragraph) indicate that it is not characteristic of human speech production. The perceived regularity of speech rhythm often arises from listener expectations in perception rather than from strict periodicity [36, 37, 38, 39].

Recent large‐scale work has examined rhythm on longer temporal windows through IUs [40]. IUs are coherent chunks of speech defined by fundamental frequency and other acoustic cues and play a crucial role in theoretical phonology [41]. To determine IUs in speech recordings from various languages, Inbar and colleagues [40] automatically assigned acoustic boundary strength scores for each word using fundamental frequency, intensity, and durational measures. IUs have been assumed to be universal building blocks of human speech [42]. They may sometimes coincide with IOIs, but they crucially involve several other acoustic dimensions, such as fundamental frequency. IOIs, as measured here, are defined by pauses and do not make reference to other acoustic cues. IOIs may contain one or multiple IUs. Inbar et al. [40] identified a low‐frequency rhythm around 0.6 Hz across more than 40 languages, proposing IUs as universal cognitively and linguistically grounded organizational units. While this study is one of the most comprehensive to date concerning cross‐linguistic comparisons and moved the field forward regarding similarities across languages, it remains tied to human‐specific linguistic segmentation at the word and phrase levels, which are impossible to adapt to nonhuman animals. Despite extensive research on fine‐grained linguistic rhythms in controlled experimental settings, cross‐linguistic analyses of larger temporal units such as IPUs and IOIs remain scarce, particularly outside of Western, educated, industrialized, rich, and democratic (WEIRD) populations [43]. Such analyses are essential to test whether universal timing patterns exist across human languages or whether they emerge only under specific ecological or communicative conditions.

### 1.5 Motivation for Our Methodology

Existing approaches to human speech rhythm typically rely on representational assumptions or language‐specific segmentation units, which limit cross‐species comparability. In our study, we analyze human speech data by quantifying temporal organization directly from the distribution of speech and pauses in the acoustic signal, without inferring underlying linguistic or cognitive representations. This methodology parallels recent work in animal communication research. A selection of rhythm measurements from studies on nonhuman animals following the same methodology is given in Table 1 for comparison. By situating our novel human data in a comparative framework with previously reported data on nonhuman animals, we hope to offer a broader perspective on where human rhythms are placed with respect to other species and discuss biomechanical or physiological constraints that shape timing in vocal behavior [5, 10, 11].

**TABLE 1:** Rhythmic parameters in animal species using the same methodology as for this study.

Class	Species	IOI duration [s] mean	CV mean	CV median	Beat [Hz] mean or range	Call	Source
Amphibia	Eastern sedge frog		0.91^a^	0.83^a^	0.8	Chorus	[7]
Amphibia	Wallum sedge frog		0.84^a^	0.73^a^	0.8	Chorus	[7]
Aves	Skylark	0.18^a^	1.3^a^	0.7^a^	6.9^a^	Flight song	[30]
Osteichthyes	Brown meagre	2.48^a^	0.18^a^	0.16			[9, 21]
Osteichthyes	*Scorpaena* spp.		0.8		0.7		[21]
Mammals	Cape fur seal	0.32	0.3		2.9		[44]
		0.37	0.08		3.2		[44]
Mammals	*Carollia perspicillata*	0.04	0.2			Isolation calls	[25]
		0.06	0.8		9.8–43.2	Echolocation	[45]
Mammals	*Desmodus rotundus*	0.08	0.6		7.4–30.5	Echolocation	
Mammals	*Glossophaga soricina*	0.03	0.6		21.9–38.9	Echolocation	
Mammals	*Lonchorhina aurita*	0.09	0.5		4.8–19.0	Echolocation	
Mammals	*Molossus molossus*	0.12	0.3		5.2–12.5	Echolocation	
Mammals	*Myotis nigricans*	0.08	0.3		10.2–18.2	Echolocation	
Mammals	*Phyllostomus hastatus*	0.06	0.5		10.4–22.5	Echolocation	
Mammals	*Pteronotus parnellii*	0.06	0.4		10.4–27	Echolocation	
Mammals	*Rhynchonycteris naso*	0.05	0.2		15.1–24.2	Echolocation	
Mammals	*Saccopteryx bilineata*	0.08	0.2			Isolation calls	[25]
		0.12	0.3		3.7–13	Echolocation	[45]
Mammals	*Saccopteryx leptura*	0.06	0.4		13.4–32.4	Echolocation	
Mammals	Sperm whale	0.46	0.1			Click coda	[25]
Mammals	*Thyroptera tricolor*	0.009	0.2		86–144.4	Echolocation	[45]

*Note*: Various rhythm parameters have been calculated for more species (see, e.g., [11]). Here, we limit ourselves strictly to studies investigating acoustic communication using the same methodology as our study. Acoustic measures contain mean IOI durations, the coefficient of variation (CV) as a mean or median, and isochronous beats in hertz as mean or range. Echolocation call sequences only considered the slower and rhythm‐wise more consistent search phase; the same is true for the click coda of the sperm whale.

^a^The data in this table were calculated from the available data in the open‐access dataset.

Our study aims to study human language by analyzing data from a sample of 48 languages spoken on all inhabited continents and covering 30 different genealogical families. Our data come from a corpus of natural speech data, from which recordings of narrative speech genres were included in our sample (see Section 4).

By focusing on the timing of IOIs (IPUs and their intervening pauses), we examine rhythmic organization at a slower temporal scale than is usually considered in linguistic rhythm studies.

We further evaluate how well these sequences can be described by an underlying isochronous model, by first calculating isochronous rhythms for a given sequence (sequence refers to a span of minimally three successive IOIs that are not interrupted by very long pauses; see Figure 1B and a detailed definition of what we consider a sequence in Table S1) and then calculating the so‐called beat precision (BP), a goodness‐of‐fit value, indicating how well a biological sequence follows the theoretical isochronous model sequence [22, 30]. BP provides a measure that again can be directly compared across taxa. Isochronous rhythms in speech production are often assumed to be absent from human language especially when looking at shorter temporal units such as intervals between linguistic syllables [37, 38]. However, given the consistent patterns found in Inbar et al. [40], we expect slower rhythms to show a high degree of regularity similar to nonhuman animals.

## 2 Materials and Methods

### 2.1 Data Preparation

Our study uses data from the DoReCo corpus v2.0 [46]. DoReCo contains speech data with time‐aligned transcriptions and annotations originating from language documentation collections covering 53 culturally and structurally diverse languages from all inhabited continents. The majority of speakers in DoReCo represent non‐WEIRD populations [47], as well as endangered, understudied, or low‐resource languages. The core datasets in DoReCo include manually verified alignments of words and silent pauses, with the latter coded based on (i) the presence of silence in the acoustic signal that cannot be attributed to articulatory processes, such as the closure phase of stop consonants, and (ii) perceptible disruptions of the speech flow. For our study, we used the core datasets from the word‐level CSV files in DoReCo 2.0 (see [48] for more details on corpus creation principles and the manual and automatic steps involved in creating the time alignments). IPUs were defined as sequences of speech uninterrupted by silent pauses (see Figure 1C) and calculated automatically from the corpus based on the time alignments provided for each word and pause unit. The onset of an IPU is the beginning of speech, that is, the onset of the first word after a silent pause as annotated in DoReCo. An IOI comprises one IPU and the following pause (in cases when the pause exceeded the threshold for pause duration, the respective IOI was discarded, and the following IPU was considered the start of a new sequence). Beats are exactly these speech onsets (for definitions of terms, see Table S1). Beat Precision (BP) is then defined as how well the time configuration of beats fits an idealized isochronous rhythm. Data were filtered to include only monological texts with personal and traditional narratives, excluding recordings of conversations or stimulus retellings. We further excluded intervals consisting entirely of filled pauses, false starts, or other content annotated with the label “<< >>” for unusual discourse events in the corpus [48]. We excluded one language (Sümi) for which only a handful of data points remained after filtering. In the end, 48 languages remained in our sample (Figure 1A). An overview of these languages, their genealogical affiliations, and the number of IOIs is provided in Table 2. Language‐level information on longitude, latitude, and genealogical family was obtained from Glottolog [49]. Note that languages are not spoken in single locations but in larger areas. The dots in Figure 1A are intended to give an indication of where approximately a language is spoken, following Glottolog [49]. Preprocessing, analysis, and plotting were performed in R [50] using RStudio [51] and Praatpicture [52].

**TABLE 2:** Languages included in our sample with unique language identification code (“Glottocode”), top‐level family as indicated in Glottolog, number of speakers (after filtering), number of files (after splitting), number of IOI units, and references for individual datasets.

	Language	Glottocode	Family	Speakers	Files	Sequences	IOI units	Source
1.	Anal	anal1239	Sino‐Tibetan	8	21	225	3059	[53]
2.	Arapaho	arap1274	Algic	4	10	132	2650	[54]
3.	Asimjeeg Datooga	tsim1256	Nilotic	13	40	84	1896	[55]
4.	Baïnounk Gubëeher	bain1259	Atlantic‐Congo	5	13	136	3102	[56]
5.	Beja	beja1238	Afro‐Asiatic	5	55	95	3548	[57]
6.	Bora	bora1263	Boran	6	9	176	2403	[58]
7.	Cabécar	cabe1245	Chibchan	6	12	42	856	[59]
8.	Cashinahua	cash1254	Pano‐Tacanan	3	7	127	2787	[60]
9.	Daakie	port1286	Austronesian	11	16	83	2188	[61]
10.	Dalabon	ngal1292	Guwinyguan	3	6	88	949	[62]
11.	Dolgan	dolg1241	Turkic	6	8	71	2175	[63]
12.	English (Southern Engl.)	sout3282	Indo‐European	2	4	64	1205	[64]
13.	Evenki	even1259	Tungusic	22	35	256	3222	[65]
14.	Fanbyak	orko1234	Austronesian	8	16	132	1490	[66]
15.	French (Swiss)	stan1290	Indo‐European	8	8	129	1870	[67]
16.	Goemai	goem1240	Afro‐Asiatic	3	9	57	1656	[68]
17.	Gorwaa	goro1270	Afro‐Asiatic	3	5	39	1315	[69]
18.	Gurindji	guri1247	Pama‐Nyungan	3	13	119	1494	[70]
19.	Hoocąk	hoch1243	Siouan	9	18	155	2402	[71]
20.	Jahai	jeha1242	Austroasiatic	3	9	105	2235	[72]
21.	Kakabe	kaka1265	Mande	4	9	48	2451	[73]
22.	Kamas	kama1351	Uralic	1	26	277	3747	[74]
23.	Komnzo	komn1238	Yam	10	17	60	2232	[75]
24.	Lower Sorbian	lowe1385	Indo‐European	4	15	157	2143	[76]
25.	Mojeño Trinitario	trin1278	Arawakan	5	8	134	1922	[77]
26.	Movima	movi1243	Isolate	5	12	172	2747	[78]
27.	Nafsan (South Efate)	sout2856	Austronesian	13	30	169	2704	[79]
28.	Nisvai	nisv1234	Austronesian	8	12	57	2001	[80]
29.	Northern Alta	nort2875	Austronesian	2	3	101	1547	[81]
30.	Northern Kurdish	nort2641	Indo‐European	2	5	34	1568	[82]
31.	N‖ng	nngg1234	Tuu	6	13	178	1939	[83]
32.	Pnar	pnar1238	Austroasiatic	4	6	48	1429	[84]
33.	Resígaro	resi1247	Arawakan	3	18	117	3076	[85]
34.	Ruuli	ruul1235	Atlantic‐Congo	3	3	35	694	[86]
35.	Sadu	sadu1234	Sino‐Tibetan	6	13	73	1663	[87]
36.	Sanzhi Dargwa	sanz1248	Nakh‐Daghestanian	5	9	53	1114	[88]
37.	Savosavo	savo1255	Isolate	7	7	86	2587	[89]
38.	Svan	svan1243	Kartvelian	9	28	89	2137	[90]
39.	Tabaq (Karko)	kark1256	Nubian	4	16	139	3238	[91]
40.	Tabasaran	taba1259	Nakh‐Daghestanian	2	5	64	1286	[92]
41.	Teop	teop1238	Austronesian	11	11	117	2473	[93]
42.	Texistepec Popoluca	texi1237	Mixe‐Zoque	1	7	230	2464	[94]
43.	Totoli	toto1304	Austronesian	7	10	154	1961	[95]
44.	Urum	urum1249	Turkic	30	129	290	2817	[96]
45.	Vera'a	vera1241	Austronesian	7	8	80	2166	[97]
46.	Yali (Apahapsili)	apah1238	Nuclear TNG	2	3	66	980	[98]
47.	Yongning Na	yong1270	Sino‐Tibetan	1	6	80	1905	[99]
48.	Yucatec Maya	yuca1254	Mayan	6	10	104	1906	[100]
	Sum			299	753	5527	101,399	

### 2.2 Analysis

The basis of our analysis consisted of individual files from the DoReCo corpus. These files contained recordings of spoken narratives, often spanning several minutes. Shorter pauses occur naturally between connected speech units (grouped into *sequences*), while unusually long pauses could be due to other reasons. We automatically determined sequence cuts in a data‐driven way, at silent pauses exceeding a certain threshold (Figure 1C). The threshold was calculated conservatively as the 99th percentile of silent pauses across the entire dataset (more than 100,000 silent pauses). The obtained duration of silent pauses used to define the start of a new sequence turned out to be 3.36 s of silence (Figure 1C provides an example of two sequences that are separated by a pause that is longer than the cutoff; Figure S1B displays the distribution of all pause durations). This means that pauses longer than 3.36 s were considered the start of a new sequence and not part of the preceding one. Only sequences containing a minimum of three IOIs were considered for our rhythm calculations, as rhythm metrics cannot be reliably calculated with sequences containing fewer observations [25]. Rhythm analysis was performed with the shiny app RANTO (https://github.com/LSBurchardt/R_app_rhythm, code version from October 2024) [23, 25, 30]. We calculated IOI beats based on the median IOI duration in a sequence transformed into a frequency:

(1) $$\mathrm{IO}I_{\mathrm{Beat}}=\frac{1}{\underline{\mathrm{IOI}}}.$$

The number of sequences analyzed for a given speaker varies across recordings. Consequently, the IOI beat data points are independent of each other to varying degrees, a fact that has to be taken into account when interpreting the results. BP values per sequence were calculated following Burchardt et al. [30] (Equation 2), with the adjustments in Equation (3) [22]:

(2) $$\mathrm{BP}=\frac{\left|\Delta \right|}{\Delta_{\max}},$$

(3) $${\mathrm{BP}}_{t}=0.5-\left|\mathrm{BP}-0.5\right|.$$

BP indicates how well a given isochronous model fits the raw IOI sequence. It does so by measuring the deviation of each onset from the closest theoretical beat, relative to the maximum possible deviation (half a beat length). This produces a value per element between 0 and 1. These values are then summarized per sequence and transformed according to Equation (2), since an average value of 0.1 per sequence would indicate the same very good fit as an average value of 0.9, with a phase shift. After transformation, a value of 0.9 would also be 0.1. Consequently, all values range from 0 to 0.5, where 0 represents a perfect fit and 0.5 a highly variable fit across a sequence. While BP is still a method under development, we find it to be a useful indicator within the context of this study, since it provides us a tool to quantify the deviation from a perfect isochronous rhythm, since a perfect isochronous rhythm does not exist in nature.

### 2.3 Use of AI Tools

When writing this article, the authors used ChatGPT (GPT‐5.1), Grammarly (Grammar Checker, up to version 8.934.0), and DeepL solely to improve clarity, grammar, and readability following the guidelines of the German Research Council (https://www.dfg.de/resource/blob/289676/230921‐statement‐executive‐committee‐ki‐ai.pdf). The authors are solely responsible for the accuracy and integrity of the scientific content.

## 3 Results

**FIGURE 2:**
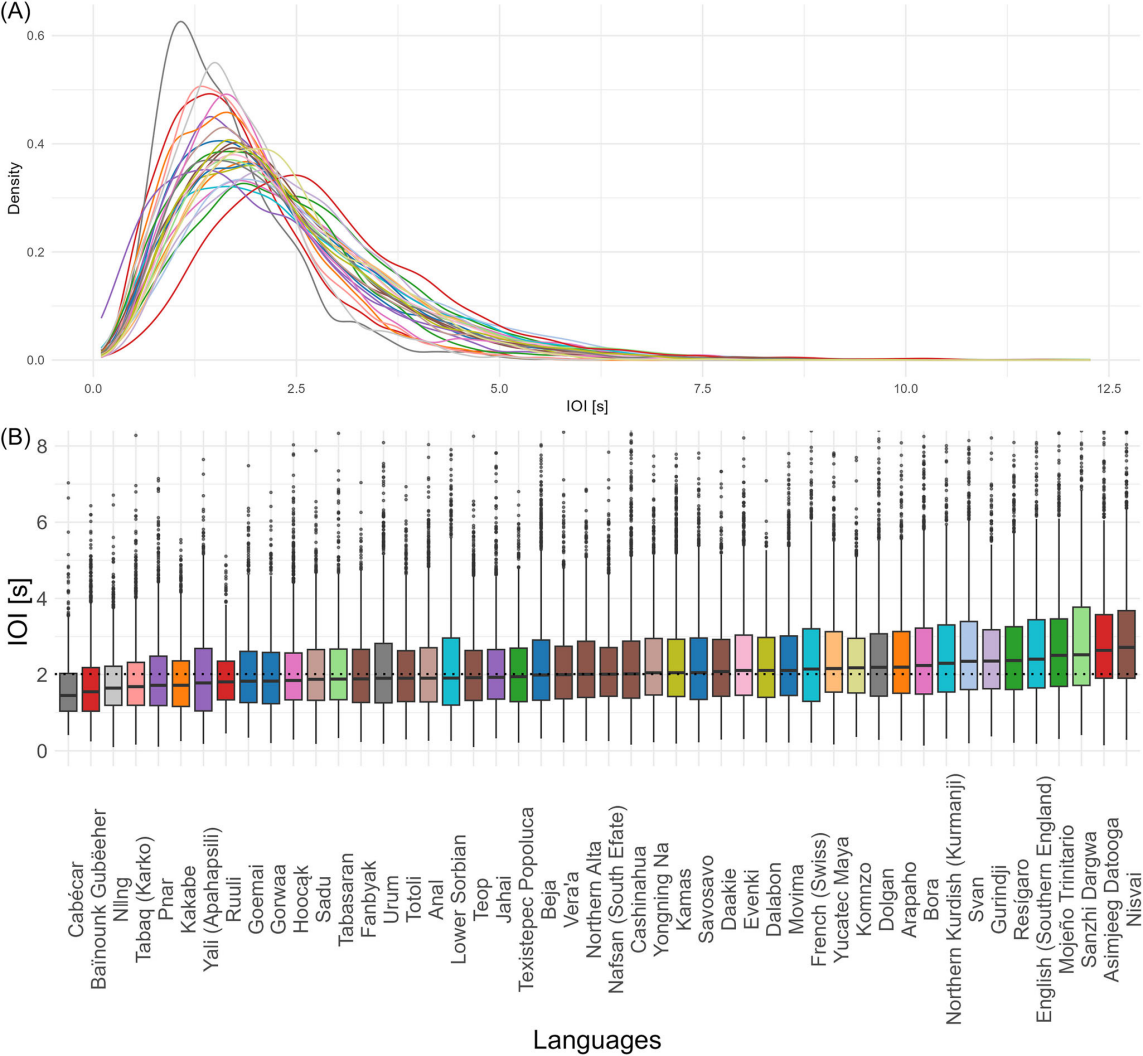
Interonset interval (IOI) durations are similar across languages and language families. (A) Density plots of intervals of all 48 languages. Colors represent language families. (B) Boxplot of IOI durations for all languages, sorted by median duration. Median IOI durations and variability look similar for most languages. The median IOI duration across all languages of 2.02 s is depicted as a horizontal dotted black line.

We analyzed a total of 5527 sequences consisting of 104,547 IOIs produced by 299 speakers across 48 languages (Figure 1; Table 2). After splitting sequences at silent pauses above the threshold and excluding very short sequences (less than three IOIs; in total 3.01% of the data were removed), we ended up with 101,399 IOIs. The results show consistent IOI durations across all languages, with a slightly right‐skewed unimodal distribution (Figure 1D). We find a median of approximately 2 s, corresponding to a rhythm of 0.5 Hz. Based on the intervals, we calculated the coefficient of variation (CV) per sequence (Figure 1E), which shows a median of 0.5 across the dataset. It reveals a considerable degree of similarity in IOIs between languages. The density distributions of IOIs are strikingly similar between languages (Figure 2A), given that these languages differ substantially in their phonological, morphological, and syntactic structures. The highest peak of the density plot corresponds to Cabécar, while the curve with a low peak to the right at ∼2.5 s corresponds to Asimjeeg Datooga (Figure 2A). Note that density plots are dependent on sample size and languages in our corpus differ in sample size.

Median interval durations across the 48 languages are also very similar in both shape and range (Figure 2B). Median IOI durations ranged from 1.4 s (Cabécar) to 2.7 s (Nisvai), with an overall median of 2.02 s (mean: 2.25 s). Sixty‐three percent of the analyzed languages show median IOI durations between 1.8 and 2.2 s, with linguistic families and areas being evenly distributed throughout the continuum.

**FIGURE 3:**
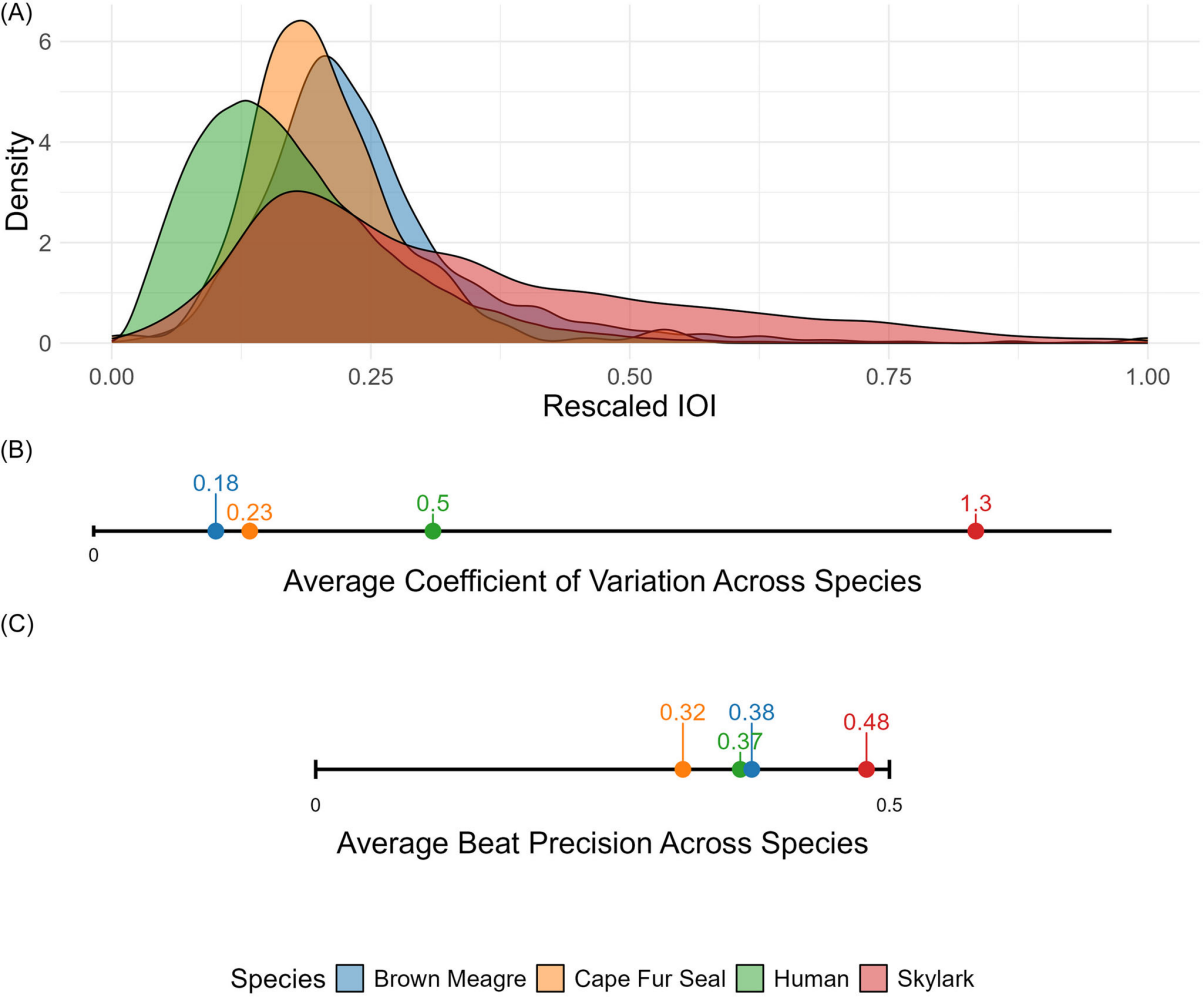
Comparison of human speech data to three other species: brown meagre (fish), Cape fur seal (mammal), and skylark (bird). All three nonhuman animal species' vocalizations have been described as having an underlying isochronous beat. The human speech data analyzed here fall within the range of previously reported values from nonhuman animals. (A) Distribution of normalized interonset interval (IOI) durations. The data have been normalized (maximum observed IOI duration = 1), as the absolute values of different species depend on factors such as body size. (B) Comparison of coefficients of variation (CVs) as a mean of CV values per sequence. (C) Comparison of beat precision (BP) values. BP values are on a scale between 0 and 0.5. Higher values for both CV and BP indicate weaker consistency in timing.

A similar pattern emerges for CV values of sequences. The distribution of sequence‐level CV values is unimodal, with a mean and median CV value of 0.5, showing no clear skew in either direction. CV values are largely similar across most languages, with outliers corresponding to languages with small sample sizes or few speakers (Yali, Ruuli; see Figure S3 and Table 2). Additionally, we tested whether IOI durations could be affected by individual (age and gender) as well as morphological and prosodic factors, but none of them show a consistent impact on IOI duration (see Supporting Information for more details, Figure S4). IOI distributions were unimodal with mostly strong peaks and CV values comparable to values found in animal sequences (see Table 1, Figure 3, and Section 4). Furthermore, normalized IOI durations of human speech and three different animal species display comparable patterns, with distributions showing considerable overlap (Figure 3A). The three species were selected according to two criteria: they represent different taxa (mammals, fish, bird), and the reported values were obtained by the same methodology as our human data. Next, we tested whether an underlying isochronous rhythm might be present, that is, whether IOI sequences could be described by an underlying isochronous beat.

We calculated beat frequencies based on the median interval per sequence (1/median IOI; see Equation 1) as an isochronous model describing the rhythm of a particular sequence. The sequence length lies between 4 and 266 IOIs, with a median of 11 IOIs (Figure 1F). Beat frequencies for the 5527 sequences across 48 languages follow a normal distribution (see Supporting Information; Figure S2A), with a mean of 0.5 Hz and a standard deviation of 0.07 Hz. Rhythms ranged from an average of 0.38 Hz in Nisvai and Asimjeeg Datooga to 0.71 Hz in Pnar and were generally similar across languages (see Supporting Information, Figure S2B). At an individual speaker level, rhythms ranged from 0.25 Hz (across five sequences) to 0.8 Hz (across three sequences). On average, there were 18 sequences per speaker (median: 10, minimum: 1, maximum: 277).

**FIGURE 4:**
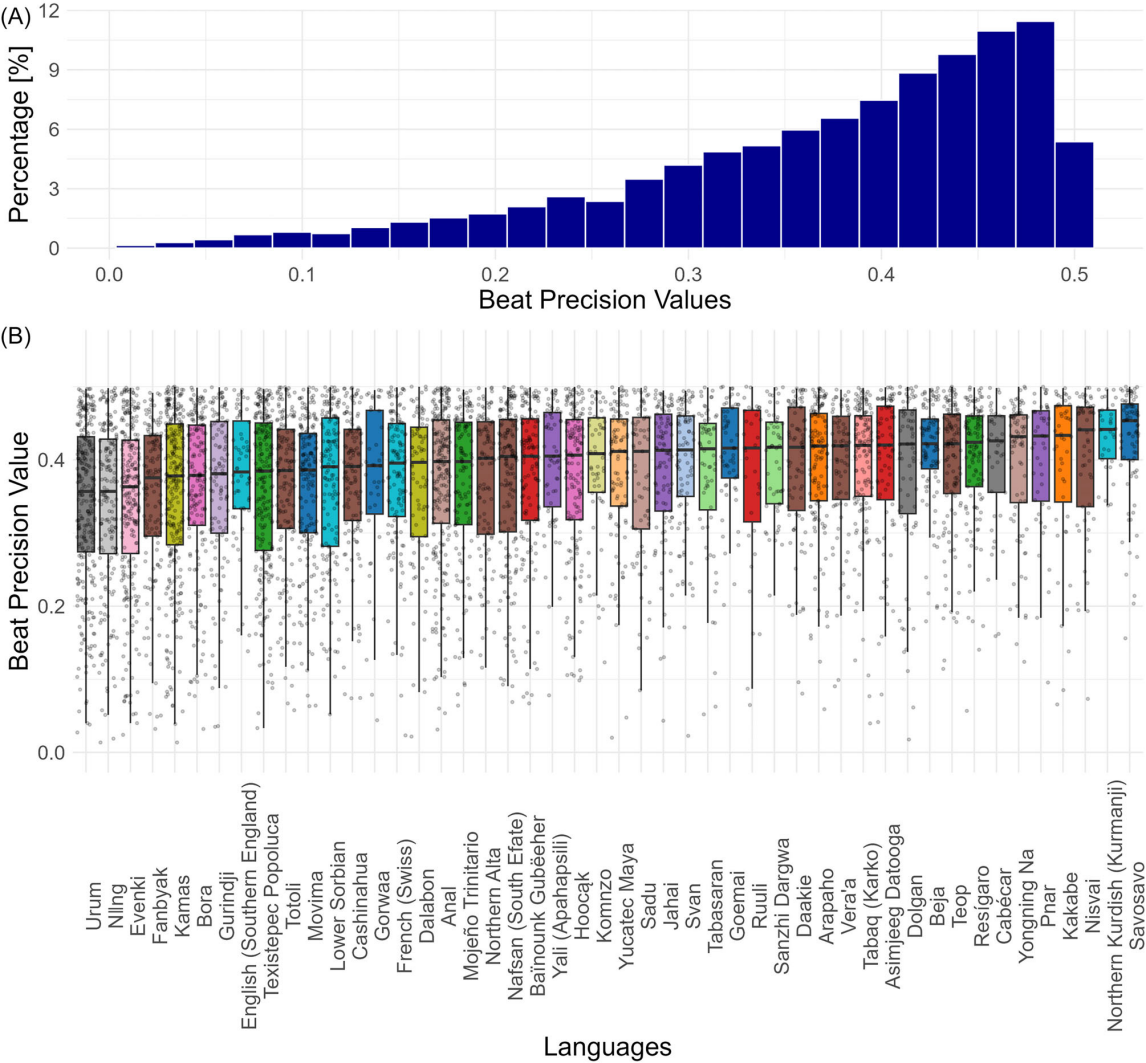
Beat precision (BP) values overall and per language and language family. (A) Histogram of BP values. While some sequences follow an underlying isochronous model, many sequences show BP values between 0.4 and 0.5, indicating a poorer fit of the underlying isochronous model to the interonset interval (IOI) sequences. (B) BP values look similar between languages and language families (indicated by different colors).

To assess how well the IOI sequences fit modeled isochronous rhythms, we calculated BP values [22, 30]. BP is reported as a summary value per sequence. A value of 0 indicates a perfect fit of the isochronous model to the IOI sequence, while a value of 0.5 indicates large deviations (i.e., strong onset jitter and interval inconsistency). BP values for individual sequences range from 0.0136 to 0.5 across all languages, indicating that some sequences follow an isochronous beat extremely well, while others are much more variable. With an overall median of 0.37, human speech fits between the Cape fur seal (*Arctocephalus pusillus pusillus*) and the brown meagre (*Sciaena umbra*) (Figure 3C), which were both previously described as very regular and isochronous [9, 44, 101]. Average BP values are similar across our 48 languages. For most languages, we find both: some sequences with small values indicating a very good fit and sequences with high values indicating no good fit (Figure 4).

## 4 Discussion

A main goal of this study was to situate human speech data in the growing landscape of rhythm descriptions across animal vocalizations. To that end, we used a data‐driven approach to segment speech and silent pauses, following a tradition in research on animal communication. We are taking a descriptive approach by presenting core rhythm measures from a diverse sample of human speech and comparing them with studies on nonhuman animal communication using the same methodology. The units of interest in our study are IOIs, which span a continuous stretch of sound and a following pause. IOI durations, CVs, and BP are presented for 48 human languages and compared with previously reported data from nonhuman animals.

To allow for a meaningful cross‐species comparison, Table 1 summarizes various rhythm parameters for 19 animal species across various taxa from recent studies using the same methodology applied to the human speech data presented here with regard to the calculation of IOI Beats and CV. As indicated earlier, many more animal species than are reported in Table 1, especially many arthropod species, are known to produce highly consistent rhythmic signals. The currently known “champion” is the male firefly species *Pteroptyx malaccae*, which shows coefficients of variation across flashing displays of as low as 0.004 [10, 102].

Mean IOI durations range from 0.009 to 2.48 s (Table 1) and mean CVs from 0.08 to 1.3. For human language, we found a median IOI duration of 2.02 s and a median CV of 0.5 across our sample. These values can be interpreted as a relatively slow rhythm with a moderate degree of variability. While human speech rhythms at this level are among the slowest observed rhythms, the CV as an independent measure of variability within a sequence is comparable to that of many nonhuman animals (Table 1, Figure 3B). With an average CV of 0.5, the human CV compares to some echolocation call sequences of different bat species (where only the slower, consistent search phase was considered; see [45]). At one end of the variability spectrum from this limited group of species (see [10, 11] for more examples), barks of adult Cape fur seal females have a very low CV of 0.08 [44], and the click coda of a female sperm whale shows a CV of 0.14 [25]; both are discussed in the literature as highly rhythmic and stereotyped, consisting of only one element type that is repeated successively. At the other end of the spectrum is the complex and long flight song of skylarks (*Alauda arvensis*) (CV = 1.3 [30]), which is made up of several motifs and up to 300 different syllables (in the biological sense, the term syllable is often defined with respect to vocalizations delineated by silent pauses) per individual, giving rise to enormous variability in syntactic combinations and, therefore, to high variability in intervals [103]. Humans also have large variability in the size of speech chunks and their combinatorial possibilities; it is therefore worth noting that the CV in this specific skylark flight song dataset was more than double the CV for human speech. Nevertheless, the analyzed sequences of skylark flight songs were mostly considerably longer than the analyzed speech sequences, which most likely increases variability.

Next to a visual comparison of CV values, Figure 3 shows normalized IOI durations for three representative species from different animal classes (fish brown meagre, Cape fur seal, skylark) next to human data from our sample. The figure illustrates that rhythmic variability in humans is not an outlier but falls within the range of that observed for the three species.

The next and most fine‐grained level of comparison concerns BP. Single BP values of sequences in our sample of human speech ranged from 0.0136 to 0.5, with a median of 0.37 (Figure 4). These results indicate that while some sequences closely align with an underlying isochronous rhythm, many sequences deviate from isochrony, sometimes very strongly. Nevertheless, especially the mean value fits well within that of other animal species, such as the Cape fur seal whose barks are described as very stereotyped [101] and have been described as isochronous (mean BP of 0.32; Figure 3) [44]. The underwater calls of the fish brown meagre have also been described as approximating an isochronous beat, and here the mean BP is even larger than that for the human speech dataset (mean BP = 0.38; Figure 3C) [9, 21]. It is important to interpret these findings with caution, as BP analysis is still under active development [22]. No matter how much variability in BP might still be considered as being close to an isochronous model (a debate we are not participating in in this study), the human data are no outlier and fit well in the range of BP found in other species.

Similar to other complex behaviors, such as vocal learning [104], rhythmicity might be better understood as a continuum or modular system [22] rather than as a binary trait. Our study adds a new data point to the ongoing effort for increased interspecies comparability to uncover the evolutionary origins of rhythms in communication signals [23], showing an example that, despite a strong unimodal distribution of IOI durations, does not universally conform to isochronous models, but also does not fall outside the range of cross‐species rhythmic organization. It is, however, important to note that nonhuman animal acoustic communication signals differ immensely in production mechanisms, functions, and environmental constraints from human speech. The underlying mechanisms governing rhythmic signal production are diverse. For example, birds use multiple inflatable air sacs and a syrinx to produce vocalizations, whereas humans rely on lungs and the larynx, while aquatic animals transmit sound waves through water, a medium distinct from air.

In human speech, the observed variation across the 48 languages still warrants further discussion. It may be influenced by a range of linguistic and speaker‐related factors. Physiological factors including body height and lung volume may be among the underlying reasons. Certain physiological features are closely tied to genetic and environmental factors; for example, populations living at high altitudes, such as in mountainous regions, generally exhibit adaptations in lung volume [105]. We did not include physiological or altitude data as factors in our study as we did not have access to the relevant metadata for all speakers. Another factor to acknowledge is that the number of analyzed sequences per speaker varied substantially. It is also sometimes impossible to disentangle individual speaker rhythms and language rhythms when data from a limited number of speakers are available, as in the case of Kamas. Kamas showed a median IOI beat of 0.48 Hz, but only recordings of a single speaker were included in our sample, and the language is now extinct. Lastly, the topics and genres of our recordings (e.g., personal narratives, traditional narratives, serious vs. comedic topics) may also have influenced rhythmic structure and could not be controlled for in this study. Many recording traditions, particularly for oral/nonliterate cultures, include narrative registers such as ritual storytelling, oratory, praise‐singing, or other performative speech that sit on a continuum with song and poetry rather than being clearly separable from them [106]. Such registers are often characterized by regular phrasing, metrical templates, or entrainment to a shared beat, features known to increase isochrony in both music and poetic recitation. Because the exact genres vary across recordings and across languages in our sample, sequences of this kind may be overrepresented in some languages and absent in others, contributing to the spread of BP values we observe. This would be consistent with our finding that high isochrony is the exception rather than the rule across the sample as a whole (as indicated by the median BP values): rather than reflecting a general property of speech rhythm, strong isochrony may emerge specifically when a recording leans toward the performative or song‐like end of the narrative spectrum. Future work explicitly coding recordings by genre would help disentangle whether this is indeed a genre effect or a genuinely language‐specific rhythmic property.

The unit of our study was the IOI, that is, uninterrupted stretches of speech and a following silent pause. At this level, languages are far less heterogeneous than at smaller units such as words or syllables (in the linguistic sense, [37]). Our findings of homogeneity in IOI durations align with previous reports on IUs. Based on a sample of 48 languages that had some overlap with but was not identical to our sample, Inbar et al. [40] found that IUs appear at a low‐frequency rhythm, with a prominent peak at about 0.6 Hz. The study revealed little variation in this IU rate across sex and age groups, and the cross‐linguistic variation in IU rate did not stem from cross‐linguistic variation in syllable rate. The crucial difference between IUs and IOIs is that the former are embedded in language‐specific phonologies, forming part of the prosodic hierarchy [41], and have no clear equivalent in nonhuman animal vocalization. IOIs, on the other hand, are defined in terms of successive vocalized and pause intervals that constitute universal building blocks of both human and nonhuman communication and thus allow for meaningful cross‐species comparisons.

Another important feature of IOIs is that unlike IUs, they always contain both stretches of speech and pauses. Previous studies on speech pauses often noted a bimodal distribution for pause durations, with shorter pauses occurring after simple clauses and longer pauses after more complex phrases [107]. However, this does not translate into a bimodal distribution for IOI durations in our data (Figure 1D). We also did not find this bimodal distribution in pause durations between IPUs (see Supporting Information, Figure S1).

Our study included data from recorded monologues and no recordings of conversations. This decision was informed by two considerations. First, the DoReCo corpus primarily comprises recordings from non‐WEIRD populations [47], where oral storytelling plays a central role in cultural transmission. These communities often lack the writing traditions characteristic of modern Western societies. Storytelling is a fundamental aspect of human communication [108], and therefore, recorded monologues can be considered a natural human vocal signal. Second, our comparison with nonhuman animal sounds is focused around self‐communication (echo location, but also directed song without an immediate response), apart from two amphibian examples that called in a chorus setting (Wallum sedge frog [*Litoria olongburensis*] and Eastern sedge frog [*Litoria fallax*]; see Table 1) in order to include at least two amphibians. This is also due to the fact that the employed methodology works well on such data but is not well tested for multi‐individual communication. We would speculate to find a higher degree of rhythmic variability for human conversational data.

It stands to reason that our findings regarding IOI durations and rhythms in human and nonhuman animals can be explained by various factors, since the organisms studied differ considerably in body size/shape, anatomical characteristics, and their social behaviors and ecological niches. One key factor, though not the only one, that may explain consistencies in IOIs across nonhuman mammals and humans is respiratory rhythm. Respiration is a fundamental driver of speech production, providing the expiratory airstream for sound production, separated by brief periods of inhalation [109]. The temporal scope of respiratory cycles may set an upper boundary for continuous vocalization.

Breathing cycles in humans are approximately 4 s on average, with great flexibility. Breath groups, that is, speech events occurring on exhalation in spontaneous speech, have been reported to range between 0.9 and 14 s or between 0.3 and 12.6 s [110, 111], broadly matching the range of IOI durations observed in our data. In principle, there may also be two or more speech units within a breath group, since not every pause goes hand in hand with inhalation. Drawing final conclusions for the age group, body size, and diversity represented in our sample is difficult, however, as cross‐linguistic data on speech breathing cycles are largely absent from the literature. Most existing data on breathing derive from controlled laboratory recordings involving participants from WEIRD populations performing reading tasks or constrained speaking activities. In contrast, speakers in the DoReCo corpus represent a substantially more diverse population sample, encompassing a broader range of spoken languages, age groups (the median age of our sample is 56 years), and cardiovascular health, among other factors. This suggests that physiologically constrained breathing cycles are one possible explanation for the cross‐linguistic similarities in IOI durations. Many nonhuman animals discussed here have lungs (all mammals), but could have additional anatomical structures, for example, birds have air sacs, and frogs can additionally breathe through their skin but mostly produce sounds with airflow from the lungs. The brown meagre, included for comparison, has an air‐filled organ, the swim bladder, and some sonic muscles acting on it to produce sound [9].

While respiratory rhythm may account for consistencies across several species, a further constraint specific to humans is working memory capacity, which typically diminishes after 2–3 s [11]. This temporal window aligns closely with the average IOI duration of about 2 s found here. Working memory, in the sense of holding and manipulating information over short delays for immediate use, is not a uniquely human phenomenon; it is also known for nonhuman animals. However, working memory capacities (how many items and how long they can be hold) may differ across species (e.g., see [112, 113]).

The exact relation between IOI durations and working memory constraints requires more attention in future studies.

In conclusion, we report that by adopting a unified, data‐driven approach to temporal organization in communication, IOI durations in 48 diverse languages from all six inhabited continents were found to cluster around 2 s, showing degrees of rhythmic variability comparable to nonhuman animals. Our study emphasizes comparability and shared constraints across humans that may arise from common physiological and biomechanical mechanisms. The comparative framework situates human communication within the broader landscape of biological rhythms, offering insights into how timing organizes behavior across species. Future work should address how much variability can be tolerated when defining isochronous rhythm and extend the analysis to a broader range of nonhuman species to obtain a more comprehensive comparative perspective. It would also be valuable to incorporate additional openly available datasets that allow the calculation of IOIs, CV, and measures of BP. Finally, further within‐species comparisons across different geographic regions or continents could help disentangle species‐specific rhythmic properties from population‐level variation.

## Author Contributions

Conceptualization: Lara S. Burchardt, Ludger Paschen, and Susanne Fuchs. Data curation: Ludger Paschen and Lara S. Burchardt. Formal analysis: Lara S. Burchardt. Writing – original draft: Lara S. Burchardt (lead), Ludger Paschen, and Susanne Fuchs. Writing – review and editing: Lara S. Burchardt, Ludger Paschen, and Susanne Fuchs (first revision); Susanne Fuchs and Ludger Paschen (second revision).

## Conflicts of Interest

The authors declare no conflicts of interest

## Supporting information




**Supplementary Materials**: nyas70383‐sup‐0001‐SuppMat.docx

## Data Availability

All relevant scripts and files are stored on GitHub (https://github.com/LSBurchardt/Rhythm_across_48_languages), including the scripts for data processing, plotting, and analysis. The input data are taken from the DoReCo corpus [46].
