# Supplementary material for: Rhythm Across Species: Situating Human Speech in a Comparative Framework
Source: Ann N Y Acad Sci. 2026 Aug 31;1563(1):e70383. doi: 10.1111/nyas.70383 (PMC13528250; doi:10.1111/nyas.70383)
Supplement: Supplementary file 1 [file NYAS-1563-0-s001.docx]

**Supporting Information for**

**Rhythm Across Species: Situating Human Speech in a Comparative Framework**

Lara S. Burchardt^a,b^, Ludger Paschen^b,c^, Susanne Fuchs^b,d^

a Humboldt Universität zu Berlin

b Leibniz-Zentrum Allgemeine Sprachwissenschaft

c Universität Regensburg

d IMéRA and ILCB, Aix-Marseille University

Corresponding Author: Susanne Fuchs

Email: fuchs@leibniz-zas.de

**This PDF file includes:**

Additional Methods

Additional Results

Figures S1 to S4

Table S1

SI References

**Additional Methods**

We visualized four parameters that might account for some of the observed variations in IOI durations: age and gender (at the speaker level) and morphological synthesis and tonal/non-tonal languages (at the language level). To probe the relation between rhythm and various speaker-related and typological features, we employed several methods to generate the relevant data frames. Information on speaker age and gender (scored as male or female; no other information was available) was extracted from the metadata files included in the corpus. Languages were manually coded as tonal or non-tonal based on available descriptions in published grammars and documentation materials. To quantify the degree of morphological synthesis, synthesis scores were calculated as the average number of morphs per word type for 38 languages with morphological annotations. We show this only qualitatively.

**Additional Results**

Previous reports have documented differences in speech rates between males and females, as well as between younger and older individuals [1–4], which we can examine in our data. We see no relation between IOI duration and speaker age (Figure S5A), nor with gender (Figure S5C).

Morphological synthesis was used as a proxy of grammatical complexity, while tone and non-tone languages often differ in their word- and phrase-level prosodies, which could affect speech rhythm. Morphological synthesis was not correlated with IOI duration, either (Figure S5B). We also see no effects for the distinction between tonal vs. non-tonal languages (Figure S5D).

**Fig. S1.** (A) Histogram of IPU durations. Most IPUs are around 1 second long, but IPUs up to 6 seconds and above are also attested. The mean IPU length is 1.43 seconds. (B) Silent pause durations across the whole dataset before sequences were split, based on the raw recordings. Mean pause duration is 0.83 seconds. The figure also shows the cut-off point at the 99th quantile. Silent pauses above 3.36 seconds resulted in a sequence being terminated. That way, one recording could be split up into several sequences. See Fig. 1 in the main manuscript for an explanation of sequences.

**Fig. S2.** IOI Beats in Hz are similar across languages and language families based on Inter-Onset Intervals. (A) Density plots of IOI Beats of all 48 languages. Colors represent language families. (B) Boxplot of IOI Beats for all languages, sorted by median IOI Beat.

**Fig. S3.** Similar Coefficients of Variation (CV) values across languages and language families based on the Coefficient of Variation per sequence. CV density is slightly right-skewed, as there are many low and medium values and a few large outliers. The order is based on the ranking of IOI beats per language as illustrated in Fig. S2.

**Fig. S4.** IOI durations are similar across age groups (A), males and female speakers (C), languages with various levels of morphological complexity (B), and between languages with and without lexical tone (D). One datapoint depicts one IOI. Due to the unbalanced nature of the number of IOIs per speaker or sequence, we decided to show all IOIs instead of summarizing per speaker or sequence.

**Table S1.** Glossary of key terms used in the manuscript.

| **Pause** | In the context of this study, pause refers to a stretch of non-vocalization of any length occurring in spoken narratives. We make no assumptions about the function of pauses, but it is known that pauses may be inserted due to respiratory, physiological, planning, rhetorical, emphatic, and other reasons. In the DoReCo corpus, pauses are annotated based on (i) the presence of silence in the acoustic signal that could not be attributed to articulatory processes, such as the closure phase of stop consonants, and (ii) perceptual disruptions in the speech flow.  Vocalized disfluencies and hesitations, although sometimes referred to as “filled pauses’’ [5], do not fall under the definition of pause applied here. |
| --- | --- |
| **IPU**  **(Inter-Pausal Unit)** | In linguistics, a stretch of speech uninterrupted by a silent pause is referred to as an Inter-Pausal Unit or IPU [6]. Some definitions of IPUs require a minimum pause length, for example, 100ms [7]. However, in this study, no such threshold criterion is applied, as pause annotations in the DoReCo corpus have been manually verified [8]. IPUs are defined solely in terms of silent pauses and do not refer to syntactic structure or prosodic domains. Usually, IPUs comprise one or two intonation units. IPUs flanked by longer pauses are more likely to constitute syntactic clauses than IPUs delineated by shorter pauses [9]. IPUs may contain stretches of disfluent speech or be made up entirely of disfluencies; in the latter case, those IPUs were excluded from the analysis in the present study. |
| **IOI (Inter-Onset Interval)** | In biology and bioacoustics, IOI (Inter-Onset Interval) refers to the time interval between the onsets of successive behavioral events, such as vocalizations, movements, or neural spikes. IOI is a critical parameter in studying rhythmic patterns, temporal structure, and sequencing in animal communication and motor behaviors [10–12]. In the context of this study, an IOI is defined as spanning a pair of one IPU and a following pause. |
| **Sequence** | A span of IOIs forms the basis of calculating speech rhythm in this study. Sequences are determined as stretches of at least 3 IOIs uninterrupted by a long pause, where “long” refers to falling outside the 99% interval of pause durations observed in the corpus (Figure S1B). A single .wav file containing a speech recording may have between one and 109 IOI sequences. |
| **Syllable** | *(linguistics)* A unit of spoken language usually consisting of a vowel nucleus, optionally with surrounding consonants, that forms the domain of certain segmental processes and can bear stress or tone.  *(biology)* Vocalisations in animal communication (e.g., bird songs) that are delineated by pauses. |
| **Mora** | A unit of phonological weight; typically, a short vowel counts as one mora, while a long vowel or a vowel plus coda consonant count as two. |
| **Stress** | The relative prominence of a syllable, typically marked by greater loudness, pitch, or duration. The rules governing the position of stress within a domain may vary substantially across the languages of the world. |
| **Prosody** | The system of suprasegmental features in speech, including intonation, rhythm, stress, and timing. |
| **Beat** | In the context of this study, beat refers to vocal onset after a pause. Successive beats in a sequence determine the rhythm. |

**SI References**

1.	Fitzsimons, M., N. Sheahan, and H. Staunton. “Gender and the Integration of Acoustic Dimensions of Prosody: Implications for Clinical Studies.” *Brain and Language* 78 (2001): 94–108.

2.	Pettorino, M., E. Pellegrino, and M. Maffia. “‘Young’ and ‘Old’ Voices: The Prosodic Auto-Transplantation Technique for Speaker’s Age Recognition,” in *Proceedings of Speech Prosody 7* (2014), 135–39.

3.	Stepanova, S. “Speech Rate as Reflection of Speaker’s Social Characteristics,” in *Approaches to Slavic Interaction*, edited by N. Thielemann and P. Kosta (Amsterdam: John Benjamins, 2013),117–29.

4.	Verhoeven, J., G. De Pauw, and H. Kloots. “Speech Rate in a Pluricentric Language: A Comparison between Dutch in Belgium and the Netherlands.” *Language and Speech* 47, no. 3 (2004): 297–308.

5.	Belz, M. “Defining Filler Particles: A Phonetic Account of the Terminology, Form, and Grammatical Classification of ‘Filled Pauses.’” *Languages* 8, no. 1 (2023): 57.

6.	Paschen, L., S. Fuchs, and F. Seifart. “Final Lengthening and Vowel Length in 25 Languages.” *Journal of Phonetics* 94 (2022): 101179.

7.	Koiso, H., Y. Horiuchi, S. Tutiya, A. Ichikawa, and Y. Den. “An Analysis of Turn-Taking and Backchannels Based on Prosodic and Syntactic Features in Japanese Map Task Dialogs.” *Language and Speech* 41 (1998): 295–321.

8.	Paschen, L., F. Delafontaine, C. Draxler, S. Fuchs, M. Stave, and F. Seifart. “Building a Time-Aligned Cross-Linguistic Reference Corpus from Language Documentation Data (DoReCo).” In *Proceedings of the Twelfth Language Resources and Evaluation Conference*, edited by N. Calzolari et al. (2020): 2657–66. Marseille: European Language Resources Association. <https://aclanthology.org/2020.lrec-1.324>.

9.	Peck, N., and L. Becker. “Syntactic Pausing? Re-Examining the Associations.” *Linguistics Vanguard* 10, no.1 (2024): 223-237.

10.	Greenfield, M., and B. Merker. “Coordinated Rhythms in Animal Species, Including Humans: Entrainment from Bushcricket Chorusing to the Philharmonic Orchestra.” *Neuroscience and Biobehavioral Reviews* 153 (2023): 105382.

11.	Hersh, T. A., A. Ravignani, and L. S. Burchardt. “Robust Rhythm Reporting Will Advance Ecological and Evolutionary Research.” *Methods in Ecology and Evolution* 14 (2023): 1398–407.

12.	Rouse, A. A., A. D. Patel, S. Wainapel, and M. H. Kao. “Sex Differences in Vocal Learning Ability in Songbirds Are Linked with Differences in Flexible Rhythm Pattern Perception.” *Animal Behaviour* 203 (2023): 193–206.
